# Recent Advances in IL-13Rα2-Directed Cancer Immunotherapy

**DOI:** 10.3389/fimmu.2022.878365

**Published:** 2022-04-08

**Authors:** Karin M. Knudson, SuJin Hwang, Mondona S. McCann, Bharat H. Joshi, Syed R. Husain, Raj K. Puri

**Affiliations:** Tumor Vaccines and Biotechnology Branch, Division of Cellular and Gene Therapies, Office of Tissues and Advanced Therapies, Center for Biologics Evaluation and Research, Food and Drug Administration, Silver Spring, MD, United States

**Keywords:** interleukin 13 receptor α2, IL-13Rα2, immunotoxin, CAR (chimeric antigen receptor) T cells, gliobastoma (GBM)

## Abstract

Interleukin-13 receptor subunit alpha-2 (IL-13Rα2, CD213A), a high-affinity membrane receptor of the anti-inflammatory Th2 cytokine IL-13, is overexpressed in a variety of solid tumors and is correlated with poor prognosis in glioblastoma, colorectal cancer, adrenocortical carcinoma, pancreatic cancer, and breast cancer. While initially hypothesized as a decoy receptor for IL-13-mediated signaling, recent evidence demonstrates IL-13 can signal through IL-13Rα2 in human cells. In addition, expression of IL-13Rα2 and IL-13Rα2-mediated signaling has been shown to promote tumor proliferation, cell survival, tumor progression, invasion, and metastasis. Given its differential expression in tumor versus normal tissue, IL-13Rα2 is an attractive immunotherapy target, as both a targetable receptor and an immunogenic antigen. Multiple promising strategies, including immunotoxins, cancer vaccines, and chimeric antigen receptor (CAR) T cells, have been developed to target IL-13Rα2. In this mini-review, we discuss recent developments surrounding IL-13Rα2-targeted therapies in pre-clinical and clinical study, including potential strategies to improve IL-13Rα2-directed cancer treatment efficacy.

## Introduction

Cytokine receptor expression is tightly regulated; however, cancer cells can overexpress cytokine receptors to promote tumor development, progression, and immune evasion. Cytokine receptors are attractive cancer therapy targets given the differential expression on normal vs. tumor tissue, the capacity to alter tumor cell fitness and function *via* receptor modulation/signaling, and variety of strategies available for selective targeting.

Interleukin-13 receptor subunit alpha-2 (IL-13Rα2) is a high-affinity membrane receptor for the anti-inflammatory cytokine interleukin 13 (IL-13). IL-13Rα2 was originally considered a decoy receptor that sequestered IL-13 and inhibited signaling since: i) IL-13Rα2 has a short cytoplasmic tail and cannot signal through canonical JAK/STAT signaling pathway (1); ii) IL-13 has higher affinity for IL-13Rα2 than its other receptor, the interleukin 13 receptor subunit alpha 1/interleukin 4 receptor subunit alpha (IL-4Rα/IL-13Rα1) heterodimer (2); and iii) overexpression of IL-13Rα2 can inhibit IL-13 signaling (3). However, recent studies demonstrated that IL-13-mediated IL-13Rα2 signaling occurs *via* STAT6-independent pathways, involving activation of activator protein 1 (AP-1) and extracellular signal-related kinase (ERK), promoting tumor invasion, metastasis, and production of transforming growth factor beta (TGFβ) (4–9). Differential binding of IL-13 by IL-13Rα1 and IL-13Rα2 has been discussed elsewhere (10, 11). More recently, Chitinase 3-like-1 (CHI3L1) was also identified as an IL-13Rα2 ligand, and a membrane protein TMEM219 was shown to be involved in IL13-Rα2 signaling (12, 13). CHI3L1 binding induces activation of mitogen-activated protein kinase (MAPK), protein kinase B (PKB)/Akt, and/or Wnt/β-catenin signaling to promote TGFβ production and tumor metastasis (12–14). 

IL-13Rα2 is overexpressed in melanoma (8, 15), renal cell carcinoma (RCC) (16), adrenocortical carcinoma (ACC) (17, 18), and a variety of brain tumors (19–21). Additionally, IL-13Rα2 overexpression correlates with advanced disease and poor prognosis in colorectal carcinoma (CRC) (22), gastric cancer (23), breast cancer (24, 25), clear cell ovarian cancer (26), lung cancer (27), ACC (28), papillary thyroid cancer (29), pancreatic ductal adenocarcinoma (30), and glioblastoma (GBM) (31–33). While IL-13Rα2 is a biomarker of prognosis for many solid tumors after therapeutic intervention, research has focused on GBM. GBM is the most common and aggressive malignant primary brain tumor in humans, and the existing treatments (i.e., tumor resection, radiotherapy, temozolomide) have limited impact on patient survival (34). IL-13Rα2 is overexpressed in ~76% of GBM but is not detected in normal brain tissue, making it a highly selective immunotherapy target (32, 33, 35).

**Figure 1:**
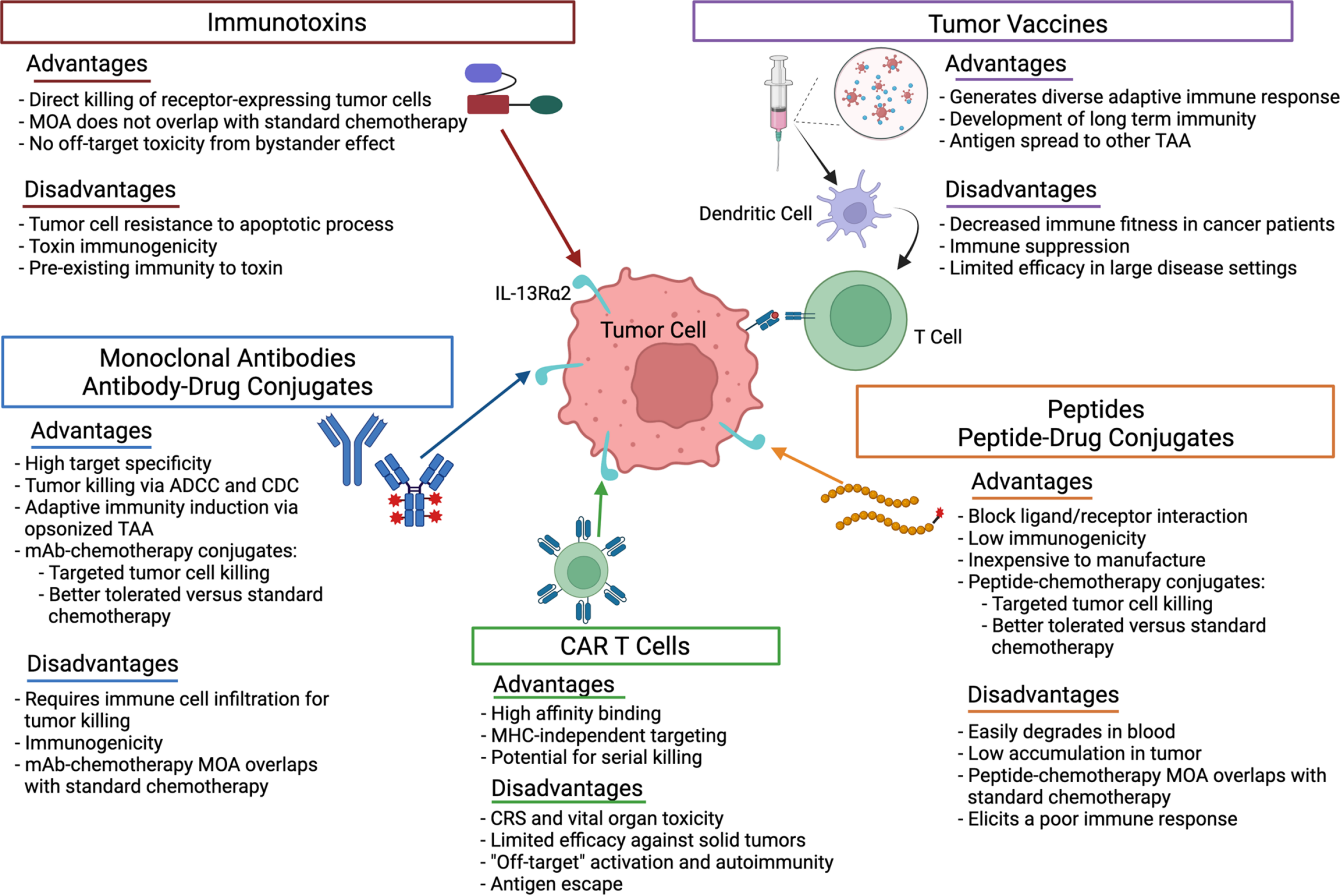
Advantages and disadvantages of the therapeutic strategies used to target IL-13Rα2 in cancer. ADCC, antibody-dependent cellular cytotoxicity; CDC, complement-dependent cytotoxicity; CRS, cytokine release syndrome; mAb, monoclonal antibody; MHC, major histocompatability complex; MOA, mechanism of action; TAA, tumor associated antigen.

In this mini-review, we focus on recent research advances for IL-13Rα2-targeted therapies. An overview of the therapies discussed and advantages/disadvantages of each are summarized in **Figure 1**. In addition, we discuss strategies to improve therapy efficacy and remaining questions in the field.

## IL-13Rα2-Targeted Immunotoxins

Recombinant immunotoxins were the first strategy to target IL-13Rα2 in cancer. These chimeric fusion proteins kill cancer cells *via* receptor binding, receptor internalization, and cleavage of the toxin moiety in the cell cytosol, which inhibits protein synthesis and induces apoptosis (36). To induce IL-13Rα2-directed killing of tumor cells, IL-13 was fused to truncated *Pseudomonas aeruginosa* exotoxin A (PE) (37) or diphtheria toxin (DT) (38).

IL13-PE (IL13-PE38QQR or cintredekin besudotox) has been used extensively in preclinical and several Phase 1-3 clinical studies. The PE38QQR exotoxin is mutated to prevent ubiquitous eukaryotic cell targeting *via* α2-macroglobulin and to enhance endoplasmic reticulum localization for production (36, 39). IL13-PE is highly cytotoxic to human solid tumor cell lines, including those derived from RCC, GBM, and head and neck squamous cell carcinoma (HNSCC) (32, 37, 40–42). IL13-PE cytotoxicity is correlated with expression of IL-13Rα2 (40, 43), and lack of expression on normal cells confers significant if not complete resistance to IL13-PE (43–45). IL13-PE treatment of orthotopic GBM and pancreatic xenografts significantly reduced tumor burden and increased overall survival (20, 45, 46). IL13-PE also decreased subcutaneous pheochromocytoma, pancreatic, and ACC xenograft tumor burden (18, 47, 48).

After intracerebral convection-enhanced delivery (CED) of IL13-PE was determined to be feasible and safe in Phase 1/2 clinical trials (49, 50), a randomized Phase 3 trial (PRECISE) with intraparenchymal IL-13PE administration was initiated in GBM patients. IL13-PE was well-tolerated but showed similar overall survival to carmustine-releasing Gliadel wafers, the only FDA-approved local treatment for recurrent GBM (51). During retrospective analysis, it was determined that only ~50% of patients had fully conforming catheters in respect to overall placement, and optimally positioned catheters had larger coverage volumes with regard to drug delivery (52, 53). Thus, efficacy of IL13-PE may have been severely constrained by delivery and diffusion issues in the PRECISE trial. Similarly, in a Phase 1 clinical trial in children with diffuse intrinsic pontine glioma, CED-delivered IL13-PE did not reach the entire MRI-defined tumor volume in any patient. Even with these constraints with drug delivery/diffusion, IL13-PE temporarily arrested disease progression in 2 of 5 patients and was well-tolerated (54). Additional clinical studies are needed to optimize distribution of CED-delivered IL13-PE.

Outside of brain malignancies, Phase 1 clinical trials using intravenous IL13-PE were initiated in RCC (55) and metastatic ACC with confirmed expression of IL-13Rα2 (56). In the ACC trial, dose limiting toxicities, including Grade 3 anemia, were observed at the higher dose (2 μg/kg) of IL13-PE. While the lower dose (1 μg/kg) of IL13-PE was well-tolerated, neutralizing antibodies were observed in all patients (56). It was not reported whether neutralizing antibodies were generated against both PE and IL-13, which will be important to identify potential toxicities. Additional clinical trials could not be performed due to lack of availability of clinical grade IL13-PE. However, to address immunogenicity issues (57), future studies should consider pre-treatment lymphodepletion, immunosuppression, or utilize a less immunogenic immunotoxin.

Two DT-conjugated IL-13 immunotoxins, DTIL13 and DT-IL13QM, also demonstrated cytotoxicity to GBM cell lines (38, 58, 59). In mice with orthotopic GBM xenografts, DTIL13 treatment significantly improved survival in a dose-dependent manner (38, 59). To improve tumor targeting and address antigen heterogeneity, bispecific immunotoxins were developed. Todhunter et al. synthesized DTAT13, which simultaneously targets overexpressed IL-13R and urokinase-type plasminogen activator receptor (60). Small subcutaneous GBM xenografts underwent regression for 40-50 days with DTAT13. Intracranial DTAT13 also had less toxicity versus DTIL13 (60). Stish et al. generated a bispecific DTEGF13 immunotoxin that targets IL-13R and epidermal growth factor (EGF) (61). Compared to monospecific immunotoxins, DTEGF13 increased cytotoxicity and reduced the growth of established prostate cancer xenografts (61). CED DTEGF13 promoted similar results in rats bearing orthotopic GBM xenografts (62). Since DT is derived from a bacterial toxin and most adults are vaccinated against diphtheria, patients may have pre-existing immunity to DT and/or quickly develop neutralizing antibodies to IL13-DT (63). Thus, similar to PE, lymphodepletion, immunosuppression, or de-immunization strategies may be needed to increase the therapeutic window of DT-conjugated immunotoxins.

## IL-13Rα2-Specific CAR-T Cells

Chimeric antigen receptor (CAR) T cell therapy is a promising treatment approach for many malignancies. First generation CARs contain a synthetic receptor typically consisting of a tumor associated antigen (TAA)-targeted extracellular single chain variable fragment (scFv), a transmembrane domain, and only the TCR CD3ζ signaling domain. Second and third generation CARs carry one or multiple costimulatory domains, respectively (64, 65). CAR T cells have produced remarkably effective and durable clinical results. Five second-generation CAR T therapies are approved by the FDA for the treatment of B-cell hematological malignancies (66). The application of CAR T cells in solid malignancies has presented many challenges, and efficacy has been limited. Regardless, IL-13Rα2-targeted CAR T cells are under investigation in six clinical trials in primary CNS malignancies and one in melanoma (*clinicaltrials.gov*, **Table 1**). Two clinical trials in primary CNS malignancies have been completed (*clinicaltrials.gov*, **Table 2**).

**Table 1:** Ongoing IL-13Rα2-targeted CAR T cell therapy clinical trials (from *clinicaltrials.gov*).

Study Title (Clinical Trial Identifier)	Study Phase (# patients)	Target Tumor	ROA	CAR T Cell (Reference)	IO Combination	Sponsor	Results (Reference)
Genetically Modified T-cells in Treating Patients With Recurrent or Refractory Malignant Glioma (**NCT02208362**)	Phase 1 (n=92)	Refractory or recurrent HGG	IT, IC or ICV	Autologous IL13BBζ TCM-enriched T cells: IL13 (E13Y) zetakine/optimized hinge/41BB/truncated CD19 (67)	None	City of Hope Medical Center	1 patient: regression of all intracranial and spinal tumors for 7.5 months (68)
IL13Rα2-CAR T Cells With or Without Nivolumab and Ipilimumab in Treating Patients With GBM (**NCT04003649**)	Phase 1 (n=60)	Resectable, recurrent GBM	ITV/ITC	Autologous IL13BBζ TCM-enriched T cells: IL13 (E13Y) zetakine/optimized hinge/41BB/truncated CD19 (67)	Ipilimumab, nivolumab	City of Hope Medical Center	No reported results
Gene Modified Immune Cells (IL13Ralpha2 CAR T Cells) After Conditioning Regimen for the Treatment of Stage IIIC or IV Melanoma (**NCT04119024**)	Phase 1 (n=24)	Stage IIIC or IV Melanoma	IV	Autologous IL13BBζ TCM-enriched T cells: IL13 (E13Y) zetakine/optimized hinge/41BB/truncated CD19 (67)	IL-2	UCLA Jonsson Comprehensive Cancer Center	No reported results
CAR T Cells After Lymphodepletion for the Treatment of IL13Rα2 Positive Recurrent or Refractory Brain Tumors in Children (**NCT04510051**)	Phase 1 (n=18)	Brain neoplasm	ICV	Autologous IL13BBζ TCM-enriched T cells: IL13 (E13Y) zetakine/optimized hinge/41BB/truncated CD19 (67)	None	City of Hope Medical Center	No reported results
Brain Tumor-Specific Immune Cells (IL13Ralpha2-CAR T Cells) for the Treatment of Leptomeningeal Glioblastoma, Ependymoma, or Medulloblastoma (**NCT04661384**)	Phase 1 (n=30)	Leptomeningeal metastases	ICV	Autologous IL13BBζ TCM-enriched T cells: IL13 (E13Y) zetakine/optimized hinge/41BB/truncated CD19 (67)	None	City of Hope Medical Center	No reported results
CART-EGFR-IL13Rα2 in EGFR Amplified Recurrent GBM (**NCT05168423**)	Phase 1 (n=18)	EGFR-amplified recurrent GBM (IDH wildtype)	IV	Autologous T cells co-expressing two CARs targeting cryptic EGFR epitope 806 and IL-13Rɑ2	None	University of Pennsylvania	No reported results
Personalized Chimeric Antigen Receptor T Cell Immunotherapy for Patients With Recurrent Malignant Gliomas (**NCT03423992**)	N/A (n=100)	Glioma	IV	Autologous CAR T cells (CAR not specified)	Anti-PD-L1	Xuanwu Hospital	(69) (IL13Rɑ2 not published)

CNS, central nervous system; CTL, cytotoxic T lymphocytes; GBM, glioblastoma; HGG, high grade glioma; IC, intracavitary; ICV, intracerebroventricular; IDH, isocitrate dehydrogenase 1; IO, immune-oncology; ITC, intracranial intratumoral; ITV, intracranial intraventricular; IV, intravenous; ROA, route of administration; TCM, central memory T cells.

**Table 2:** Completed IL-13Rα2-targeted CAR T cell therapy clinical trials (from *clinicaltrials.gov*).

Study Title (Clinical Trial Identifier)	Study Phase (# patients)	Target Tumor	ROA	CAR T Cell (Reference)	IO Combination	Sponsor	Results (Reference)
Cellular Adoptive Immunotherapy Using Genetically Modified T-Lymphocytes in Treating Patients With Recurrent or Refractory High-Grade Malignant Glioma (**NCT00730613**)	Phase 1 (n=3)	Brain and CNS tumors	IC	Autologous IL13(E13Y)-zetakine/HSV-TK CD8+ CTL (70)	None	City of Hope Medical Center	2/3 patients: positive response (71)
Phase I Study of Cellular Immunotherapy for Recurrent/Refractory Malignant Glioma Using Intratumoral Infusions of GRm13Z40-2, An Allogeneic CD8+ Cytolytic T-Cell Line Genetically Modified to Express the IL 13-Zetakine and HyTK and to be Resistant to Glucocorticoids, in Combination With Interleukin-2 (**NCT01082926**)	Phase 1 (n=6)	Stage III, IV malignant glioma	IT	Allogeneic IL13(E13Y)- zetakine/HSV-TK CD8+ CTL	IL-2	City of Hope Medical Center	No reported results

CNS, central nervous system; CTL, cytotoxic T lymphocytes; HSV-TK, HSV-1 thymidine kinase selection-suicide domain; IC, intracavitary; IO, immune-oncology; IT, intratumoral; ROA, route of administration.

First generation IL-13(E13Y) zetakine CAR T cells (IL13-zetakine CTL (cytotoxic T lymphocytes)) utilized a membrane-tethered, mutated IL-13 (E13Y) instead of scFv to redirect T cells to IL-13Rα2 and reduce cross-reactivity with IL-13Rα1. Initial studies demonstrated remarkable efficacy of IL13-zetakine CTL against human GBM orthotopic xenografts and no-cross reactivity with IL-13Rα1 (70). However, additional studies suggested IL13-zetakine CTLs do recognize IL-13Rα1-positive targets, which could lead to off-target toxicities (71–73). The first-in-human trial using intracranial delivery of first generation IL13-zetakine CAR T cells demonstrated that repeated administration was well-tolerated, and two of three patients underwent transient anti-glioma responses. Durable responses were not observed, which correlated with short CAR T cell persistence (71).

Second-generation IL13BBζ CAR T cells were genetically engineered to incorporate IL-13 (E13Y-mutated), 4-1BB (CD137), and mutated IgG4-Fc linker, which resulted in the enrichment of central memory T cells (T_CM_). In orthotopic human GBM models, anti-tumor activity and T cell persistence were significantly improved in IL13BBζ (67). IL13BBζ CAR T cells are currently in clinical trials. In one patient, multiple intracranial infusions of IL13BBζ CAR T cells over 220 days were well-tolerated, increased cytokine levels and immune cell frequencies were observed in the cerebrospinal fluid, and the patient underwent regression for 7.5 months (68). While none of the initial tumors recurred, preliminary results suggest recurrence of new tumors with reduced expression of IL-13Rα2. Thus, antigen escape may be a significant issue against the generation of durable responses.

More recently, to further reduce cross-reactivity with IL-13Rα1, Kim et al. reported the generation of YYB-103, a mutated IL-13-based CAR with multiple amino acid substitutions (E13K, R66D, S69D, and R109K) (74). YYB-103 was more selective than IL-13(E13Y) zetakine CTL for IL-13Rα2. Intracranial and intravenous administration of YYB-103 reduced orthotopic GBM xenograft tumor burden and increased survival (74).

On the other hand, Krenciute et al. constructed a panel of IL-13Rα2-specific CARs containing an anti-IL-13Rα2-scFv instead of a mutated IL-13, short or long spacer regions, a transmembrane domain, endodomains derived from costimulatory molecules CD28, 4-1BB, or OX40, and CD3ζ (75). In a murine GBM study, IL-13Rα2-CAR T cells with short spacer regions and CD28ζ, 4-1BBζ, and CD28.OX40ζ displayed potent anti-glioma activity with high specificity for IL-13Rα2 and no cross-reactivity to IL-13Rα1 (75). CD28ζ CAR T cells persisted and proliferated in the brains of glioma-bearing mice after intracranial administration (76). Importantly, mice that underwent complete IL-13Rα2-positive tumor regression were also protected against rechallenge with IL-13Rα2-negative tumors, suggesting that the IL-13Rα2-specific CARs assist in the development of diversified long-term anti-glioma immunity (76).

Given the heterogeneity of antigens on GBM tumors, multi-antigen-targeted CAR molecules may enhance efficacy and mitigate antigen escape observed with unispecific IL13BBζ CAR T cells (68). Hegde et al. discovered that 96% of patient GBM tumors expressed human epidermal growth factor 2 (HER2), IL-13Rα2, or ephrin-A2 (EphA2) (77). Co-expression of HER2- and IL-13Rα2-specific CARs enhanced T cell functionality against autologous glioma cells. Compared with individual or pooled monospecific CAR T cells, HER2/IL-13Rα2 CARs improved tumor control and survival in a human orthotopic GBM model (77). TanCAR, a CAR T cell containing a bispecific HER2-binding scFv/mutated IL-13 heterodimer CAR, also improved survival in a GBM model verses monospecific CAR T cells (78). To further improve the efficacy of TanCAR T cells, Bielamowicz et al. generated trivalent CAR T cells using a single universal tricistronic transgene to co-express CARs specific for HER2, IL-13Rα2, and EphA2 on patient T cells (UCAR T cells) (79). All three CARs were successfully co-expressed and had antigen-specific functionality. In a tumor model using patient GBM xenografts and patient-matched T cells, UCAR T cells derived from 2/2 patients decreased tumor burden and increased survival versus univalent CAR T cells. Similar results were observed for UCAR T cells derived from 1/2 patients when compared to bivalent CAR T cells (79).

## IL-13Rα2-Targeted Peptides and Inhibitors of IL-13Rα2 Signaling

Peptides targeting IL-13Rα2 can be used to block native ligand-receptor interaction/signaling and/or deliver conjugated therapeutic agents to the tumor microenvironment (TME). Compared to antibodies, peptides display lower immunogenicity, better tumoral diffusion due to low molecular weight, and are easier and inexpensive to synthesize (80). Various techniques are used to improve peptide stability in biological fluids and increase accumulation in the tumor, including the use of D-amino acids and PEGylation (80).

Using phage display technology, Pandaya et al. identified Pep-1L peptide that binds specifically to IL-13Rα2 *via* a non-competitive binding site for IL-13 (81). Intravenous Pep-1L accumulated in both subcutaneous and orthotopic GBM tumors (81–83), indicating that Pep-1L efficiently crosses the blood-brain-tumor barrier. Pep-1L binding also induced IL-13Rα2 internalization (81, 84), making it an attractive candidate to deliver cytotoxic agents to IL-13Rα2-expressing tumors. Indeed, intracranial CED of a Pep-1L-alpha particle emitter conjugate promoted GBM cytotoxicity and increased overall survival in an orthotopic murine GBM model (85). Similarly, Pep-1L-paclitaxel (chemotherapy) nanoparticle conjugates reduced intracranial glioma growth and increased overall survival (83).

Bartolome et al. used the recently elucidated structure of the IL-13/IL-13Rα2 complex to engineer a 12-mer peptide (D1) that specifically blocks the IL-13/IL-13Rα2 signaling axis (86). D1 peptide inhibited IL-13Rα2-mediated signaling to a greater extent than IL-13Rα1 signaling. D1 also significantly reduced IL-13-mediated binding, cell migration, and invasion of CRC and GBM cell lines, and increased survival in xenograft models (86).

Recent insights into the downstream signaling of IL-13Rα2 have also allowed for targeting of IL-13Rα2-mediated signaling by small molecule inhibitors. Bartolome et al. identified protein tyrosine phosphatase-1B (PTP1B) as a mediator of IL-13Rα2 signaling (87). PTP1B is overexpressed in many tumor types, and high expression correlated with reduced overall survival of GBM, CRC, and ovarian cancer patients. Use of the PTP1B inhibitor, Claramine significantly reduced tumor burden, metastasis and increased survival in mice with CRC and GBM xenografts (87).

## IL-13Rα2-Directed Monoclonal Antibodies

Many agents targeting IL-13Rα2 lack the selectivity to bind IL-13Rα2 but not IL-13Rα1. Monoclonal antibodies (mAbs) bind targets with high affinity/specificity and mediate efficacy via: i) manipulation/inhibition of critical signaling pathways required for the malignant phenotype; ii) initiation of antibody-dependent cellular cytotoxicity (ADCC); iii) complement-dependent cytotoxicity (CDC) by complement activation; and/or iv) increased presentation of opsonized antigens by antigen presenting cells (APC) (88, 89).

In order to target IL-13Rα2 but not IL-13Rα1, Balyasnikova et al. characterized a novel antibody that blocks IL-13/IL-13Rα2 interaction (90). This mAb bound IL-13Rα2 in GBM tissue and improved survival of mice with orthotopic human glioma xenografts (90). Using a similar strategy, Jaen et al. generated a D1 peptide-specific mAb that blocked IL-13/IL-13Rα2 -mediated signaling (91). Correlating with inhibition of IL-13-mediated CRC cell migration and invasion *in vitro*, the D1-specific mAb also reduced liver metastasis of CRC tumors and improved survival (91).

Like peptides, mAbs can be conjugated to other therapeutic agents. Biodistribution studies performed by Gupta et al. demonstrated that intravenous IL-13Rα2 mAb led to a time-dependent, selective accumulation of mAb in IL-13Rα2-expressing tumors (91). Accumulation was not affected by conjugation to auristatin, an antimitotic agent (92, 93). The mAb-auristatin conjugate significantly reduced melanoma xenograft growth in a dose-dependent manner, and 90% of mice underwent complete tumor rejection at the highest dose (93). Interestingly, conjugation of another IL-13Rα2 mAb to auristatin did not impact growth of certain IL-13Rα2-expressing diffuse intrinsic pontine glioma (DIPG) cell lines (94), suggesting selection of appropriate drug conjugates is critical for efficacy in different tumor settings.

## IL-13Rα2-Targeted Therapeutic Cancer Vaccines

In contrast to passive therapeutic approaches like peptides or mAb, therapeutic cancer vaccines can promote the development of a diverse, long-term immune response against TAA. Therapeutic cancer vaccines can consist of whole tumor cells, tumor cell lysate or peptides/proteins mixed with an adjuvant, viruses genetically-modified to express a TAA, or TAA-pulsed APCs (95).

The most clinically advanced IL-13Rα2-directed vaccine studies utilize peptide-pulsed dendritic cells (DCs). IL-13Rα2 peptide-pulsed DCs induce T cell responses in recurrent glioma patients (96–98). However, most recent clinical trials evaluate efficacy of DCs pulsed with IL-13Rα2 antigen and other glioma-associated peptides. Okada et al. evaluated DCs loaded with four peptides, including IL-13Rα2, in combination with Poly-ICLC adjuvant (99). Nine of 22 patients achieved progression-free status lasting at least 12 months (99). After showing favorable results in a Phase 1 trial (98), ICT-107, an autologous DC vaccine with six synthetic GBM-associated peptides including IL-13Rα2, was evaluated in a double-blinded, randomized phase 2 clinical trial in newly diagnosed patients with GBM. Overall, ICT-107 was well-tolerated and increased progression-free survival by 2.2 months (100). A Phase 3 trial of ICT-107 has been established (NCT02546102).

Vaccination with IL-13Rα2 DNA significantly reduced murine syngeneic tumor growth through induction of T and B cell responses (101, 102). Anti-tumor efficacy was enhanced with IL-13Rα2 DNA priming plus a protein/adjuvant boost consisting of the extracellular domain of IL-13Rα2 protein, CpG, and incomplete Freund’s adjuvant (IFA) (103). Human studies have not been initiated. Instead of using DNA vaccination, Pollack et al. immunized children with newly diagnosed diffuse brainstem and high-grade gliomas using three glioma-associated antigens, including IL-13Rα2, in combination with Poly ICLC adjuvant (104). Anti-glioma antigen immune responses to IL-13Rα2 were observed in 10 of 13 evaluable patients. Two patients had prolonged disease-free status after surgery (104).

## Conclusions and Future Perspectives

Over the past 20 years, IL-13Rα2 has been confirmed as an effective target for novel cancer therapies. We predict that investigation of IL-13Rα2-targeted therapies will continue in the clinic, with increased treatment of solid tumors outside of gliomas. While transient improvements in patient outcomes have been observed with IL-13Rα2-targeted monotherapies, even in hard-to-treat tumors like GBM, the overall clinical response has been underwhelming. Thus, combinatorial approaches are likely necessary for the development of robust IL-13Rα2-targeted anti-tumor responses.

Chemotherapy is considered standard of care in many indications and works with an entirely different mechanism of action compared to targeted therapies. We have combined chemotherapy with IL-13Rα2-directed immunotoxin therapy. When pancreatic tumor cell lines were treated with gemcitabine, IL-13Rα2 was upregulated, resulting into increased IL13-PE-mediated killing *in vitro* and improved survival of mice implanted with pancreatic tumor xenografts (105). Similar results were observed in oral squamous cell carcinoma preclinical models (106). Thus, chemotherapy may synergize or enhance antitumor effects through upregulation of IL-13Rα2 in low expressing tumors or through additional mechanisms yet to be elucidated. However, chemotherapy/IL-13Rα2-targeted combinations may not be effective in certain indications. For example, a correlation between temozolomide resistance and IL-13Rα2 expression has been observed in GBM (31). Other therapeutic approaches like use of histone deacetylase (HDAC) inhibitors, which can cause over-expression of some tumor-associated genes, may be a viable substitute for chemotherapy combination in resistant tumors. Fujisawa et al. reported that HDAC inhibition upregulated IL-13Rα2 and increased IL13-PE-mediated responses in pancreatic tumor models (48).

While therapeutics like IL13-PE or IL-13Rα2-mAb-chemotherapy conjugates are effective in reducing tumor burden through direct cytotoxicity, combination with other IL-13Rα2-targeted therapies such as cancer vaccines or CAR T cells may be necessary to ensure long-term responses. Supporting this, IL-13Rα2 DNA vaccine/IL13-PE combination synergized to reduce murine sarcoma and breast tumor growth *via* multiple immune mechanisms, including direct tumor killing and increased T cell tumor infiltration (102). As an alternative to using multiple IL-13Rα2-targeted therapies, to address tumor antigen heterogeneity, it may be better to combine agents that target distinct TAA. IL-13Rα2 expression declines during IL-13Rα2-targeted therapy (71), likely due to selective killing of IL-13Rα2-expressing cells and downregulation of IL-13Rα2 as a resistance mechanism to mediate tumor escape. Utilizing therapies to target multiple TAA may prevent antigen escape; however, targeting multiple TAA, especially when using bi- or multi-specific molecules, may increase on-target, off-tumor or off-target toxicities and/or lower the maximal tolerated dose. Finally, the combination of IL13Rα2-targeted therapies with checkpoint inhibitors may reduce immunosuppression within the tumor microenvironment (TME) and improve efficacy of IL-13Rα2-specific CAR T cells (107). One recent study showed a positive correlation of IL-13Rα2 and the immune regulatory protein VISTA in oral squamous cell carcinoma (108). Other checkpoint inhibitors may also be upregulated in tandem with IL-13Rα2.

In addition to identifying the combinations to improve IL-13Rα2-targeted therapies, toxicities associated with targeting IL-13Rα2 should be considered. While many IL-13Rα2-targeted therapies are engineered to reduce cross-reactivity with IL-13Rα1, some still display such cross-reactivity. IL-13Rα1, unlike IL-13Rα2, is ubiquitously expressed in humans. As such, there is a hypothetical risk of IL-13Rα1-related off-target toxicities when using IL-13Rα2-targeted therapies. To date, these toxicities have not been observed in the clinic, but close monitoring of patients is important. Most active clinical trials deliver IL-13Rα2-targeted therapies locally in the brain, so it is possible that novel toxicities may be observed with systemic or intracavital administration.

Importantly, the on-target, off-tumor effects of IL-13Rα2-targeted therapies have not been widely investigated. Certain immune cells express IL-13Rα2, including monocytes, myeloid-derived suppressor cells (MDSC), and macrophages (6, 109–111). After IL-13Rα2-targeted CAR T cell treatment, mice with orthotopic murine gliomas have a transient reduction in MDSC number in the tumor and spleen (76). IL13-PE treatment also reduced the frequency of MDSC in the tumor and/or spleen of mice bearing syngeneic HNSCC and breast tumors (102, 112). It has not been elucidated whether reduction in MDSC prevalence is due to direct killing of IL-13Rα2-expressing MDSC or cytokine-mediated reprogramming of these cells to a different phenotype. Both mechanisms are likely, as IL-13Rα2 signaling has been linked to M2 polarization of human macrophages (6, 111). While IL-13Rα2-mediated MDSC depletion would likely improve anti-tumor responses, reduction of other IL-13Rα2-expressing immune cell populations, especially those that mediate anti-tumor efficacy, may have negative effects. Thus, immune expression of IL-13Rα2 within the TME or in tumor-bearing subjects requires further elucidation.

## Author Contributions

RKP conceived, reviewed, and supervised the mini-review. KMK, SH, and MSM collected literature, wrote the review, and revised the manuscript. MSM and KMK generated the tables and figure. BHJ and SRH reviewed and edited the manuscript. All authors reviewed and approved the manuscript.

## Funding

The writing of this review was supported through the intramural funding of the Office of Tissues and Advanced Therapies, Center for Biologics Evaluation and Research, FDA.

## Conflict of Interest

The authors declare that the research was conducted in the absence of any commercial or financial relationships that could be construed as a potential conflict of interest.

## Publisher’s Note

All claims expressed in this article are solely those of the authors and do not necessarily represent those of their affiliated organizations, or those of the publisher, the editors and the reviewers. Any product that may be evaluated in this article, or claim that may be made by its manufacturer, is not guaranteed or endorsed by the publisher.
